# Association of cerebral metabolic rate following therapeutic hypothermia with 18-month neurodevelopmental outcomes after neonatal hypoxic ischemic encephalopathy

**DOI:** 10.1016/j.ebiom.2023.104673

**Published:** 2023-06-29

**Authors:** Jason Sutin, Rutvi Vyas, Henry A. Feldman, Silvina Ferradal, Chuan-Heng Hsiao, Lucca Zampolli, Lara J. Pierce, Charles A. Nelson, Sarah U. Morton, Susanne Hay, Mohamed El-Dib, Janet S. Soul, Pei-Yi Lin, Patricia E. Grant

**Affiliations:** a Fetal-Neonatal Neuroimaging and Developmental Science Center, Boston Children's Hospital, 300 Longwood Ave., Boston, MA 02115, USA; b Division of Newborn Medicine, Department of Pediatrics, Boston Children's Hospital, 300 Longwood Ave., Boston, MA 02115, USA; c Harvard Medical School, 25 Shattuck St., Boston, MA 02115, USA; d Department of Pediatrics, Institutional Centers for Clinical and Translational Research, Boston Children's Hospital, 300 Longwood Ave., Boston, MA 02115, USA; e Department of Intelligent Systems Engineering, Indiana University Bloomington, 107 S Indiana Ave., Bloomington, IN 47405, USA; f Department of Psychology, York University, 198 York Blvd., North York, ON M3J 2S5, Canada; g Division of Developmental Medicine, Department of Pediatrics, Boston Children's Hospital, 300 Longwood Ave., Boston, MA 02115, USA; h Department of Neonatology, Beth Israel Deaconess Medical Center, 330 Brookline Ave., Boston, MA 02215, USA; i Division of Newborn Medicine, Department of Pediatrics, Brigham and Women's Hospital, 75 Francis St., Boston, MA 02115, USA; j Department of Neurology, Boston Children's Hospital, 300 Longwood Ave., Boston, MA 02115, USA; k Department of Radiology, Boston Children's Hospital, 300 Longwood Ave., Boston, MA 02115, USA

**Keywords:** Therapeutic hypothermia, Hypoxic ischemic encephalopathy, Perinatal asphyxia, Neonates, Infants, Neurodevelopmental outcome, Cerebral metabolic rate of oxygen consumption, Cerebral blood flow, Diffuse correlation spectroscopy, Near-infrared spectroscopy

## Abstract

**Background:**

Therapeutic hypothermia (TH) is standard of care for moderate to severe neonatal hypoxic ischemic encephalopathy (HIE) but many survivors still suffer lifelong disabilities and benefits of TH for mild HIE are under active debate. Development of objective diagnostics, with sensitivity to mild HIE, are needed to select, guide, and assess response to treatment. The objective of this study was to determine if cerebral oxygen metabolism (CMRO_2_) in the days after TH is associated with 18-month neurodevelopmental outcomes as the first step in evaluating CMRO_2_'s potential as a diagnostic for HIE. Secondary objectives were to compare associations with clinical exams and characterise the relationship between CMRO_2_ and temperature during TH.

**Methods:**

This was a prospective, multicentre, observational, cohort study of neonates clinically diagnosed with HIE and treated with TH recruited from the tertiary neonatal intensive care units (NICUs) of Boston Children's Hospital, Brigham and Women's Hospital, and Beth Israel Deaconess Medical Center between December 2015 and October 2019 with follow-up to 18 months. In total, 329 neonates ≥34 weeks gestational age admitted with perinatal asphyxia and suspected HIE were identified. 179 were approached, 103 enrolled, 73 received TH, and 64 were included. CMRO_2_ was measured at the NICU bedside by frequency-domain near-infrared and diffuse correlation spectroscopies (FDNIRS-DCS) during the late phases of hypothermia (C), rewarming (RW) and after return to normothermia (NT). Additional variables were body temperature and clinical neonatal encephalopathy (NE) scores, as well as findings from magnetic resonance imaging (MRI) and spectroscopy (MRS). Primary outcome was the Bayley Scales of Infant and Toddler Development, Third Edition (BSID-III) at 18 months, normed (SD) to 100 (15).

**Findings:**

Data quality for 58 neonates was sufficient for analysis. CMRO_2_ changed by 14.4% per °C (95% CI, 14.2–14.6) relative to its baseline at NT while cerebral tissue oxygen extraction fraction (cFTOE) changed by only 2.2% per °C (95% CI, 2.1–2.4) for net changes from C to NT of 91% and 8%, respectively. Follow-up data for 2 were incomplete, 33 declined and 1 died, leaving 22 participants (mean [SD] postnatal age, 19.1 [1.2] month; 11 female) with mild to moderate HIE (median [IQR] NE score, 4 [3–6]) and 21 (95%) with BSID-III scores >85 at 18 months. CMRO_2_ at NT was positively associated with cognitive and motor composite scores (β (SE) = 4.49 (1.55) and 2.77 (1.00) BSID-III points per 10^−10^ moL/dl × mm^2^/s, *P* = 0.009 and *P* = 0.01 respectively; linear regression); none of the other measures were associated with the neurodevelopmental outcomes.

**Interpretation:**

Point of care measures of CMRO_2_ in the NICU during C and RW showed dramatic changes and potential to assess individual response to TH. CMRO_2_ following TH outperformed conventional clinical evaluations (NE score, cFTOE, and MRI/MRS) at predicting cognitive and motor outcomes at 18 months for mild to moderate HIE, providing a promising objective, physiologically-based diagnostic for HIE.

**Funding:**

This clinical study was funded by an NIH grant from the 10.13039/100009633Eunice Kennedy Shriver National Institute of Child Health and Human Development, United States (R01HD076258).

**Research in context**

**Evidence before this study**

While hypoxic ischemic encephalopathy (HIE) remains a major source of admissions to neonatal intensive care units (NICUs), therapeutic hypothermia (TH) has dramatically improved patient survival and quality of life. However, challenges and controversies persist with many survivors still facing neurodevelopmental impairment, and the field also grapples with the question of how—or even whether—to treat mild encephalopathy. A significant impediment to further improvements in care is that current diagnostic tools are subjective, with limited ability to evaluate HIE and response to treatment, especially for the poorly understood mild HIE, where little progress has been previously reported. Because cerebral metabolism is directly related to the pathophysiology of HIE and the therapeutic effect of TH, measuring the cerebral metabolic rate of oxygen consumption (CMRO_2_) has enormous potential to improve diagnostic and prognostic accuracy in neonatal encephalopathy. We searched *PubMed* from inception to November 17, 2022 using the search terms “cerebral oxygen metabolism” AND “therapeutic hypothermia” and only three publications were found. One was preclinical, one was our previous pilot, and one was a feasibility study with T2-Relaxation-Under-Spin-Tagging (TRUST) MRI. None investigated correlations with later outcome.

**Added value of this study**

Using near-infrared spectroscopy (NIRS) combined with diffuse correlation spectroscopy (DCS), we report a statistically robust clinical study of therapeutic hypothermia for neonatal hypoxic ischemic encephalopathy (HIE) that assesses the power of CMRO_2_ to predict neurodevelopmental outcomes. We enrolled neonates prospectively at three NICUs and found CMRO_2_ increased upon rewarming with about a tenfold greater change than cerebral tissue oxygen saturation or extraction fraction, the variables more commonly measured by clinical cerebral oximeters, indicating CMRO_2_ is a more sensitive indicator of the effects of treatment. HIE in the cohort was mild to moderate with CMRO_2_ during normothermia in the days after hypothermia predicted later cognitive and motor outcomes at 18 months of age. In contrast, conventional clinical evaluations of neurological exams, magnetic resonance spectroscopy and imaging (MRS and MRI), and cerebral oximetry had no association.

**Implications of all the available evidence**

Our results help establish CMRO_2_ as an objective, quantitative biomarker for neonatal brain injuries in HIE by linking CMRO_2_ after TH to a clinically relevant endpoint. We have proven the value of CMRO_2_ as an early, objective outcome predictor for HIE, with sensitivity to the effects of mild encephalopathy where neurodevelopmental outcomes are difficult to predict. Importantly, we introduce point of care monitoring of CMRO_2_ in the NICU as a promising paradigm for evaluation of brain injury in HIE, presented in context of the performance of existing clinical diagnostics and with mechanistic insights as to why they fall short. We also provide evidence for physiological underpinnings that bolster findings from recent clinical studies that mild HIE has greater impact on survivors than commonly recognized. This work is a foundation for further investigation into the diagnostic potential of CMRO_2_ to provide actionable information to reduce the severity of brain injuries from HIE.

## Introduction

Hypoxic ischemic encephalopathy (HIE) remains a common cause of neonatal death and long-term disability, occurring in 1.5–3 per 1000 live births.^1^^,^^2^ In high-income countries, therapeutic hypothermia (TH) for treatment of neonatal HIE significantly reduces adverse outcomes and has become the standard of care for moderate-to-severe HIE.^3^, ^4^, ^5^ However, even with TH, up to 40% of HIE survivors still suffer from neurodevelopmental impairment.^6^^,^^7^ Furthermore, the effectiveness of TH in mild HIE has not been well studied; no consensus exists for using TH to treat mild HIE despite recent evidence that survivors of untreated mild HIE have worse neurodevelopmental outcomes than healthy neonates.^8^, ^9^, ^10^, ^11^ Development of objective diagnostics, with sensitivity to mild HIE, are needed to improve selection, management, and assessment of response to treatment.

HIE occurs when a hypoxic ischemic insult impairs cerebral blood flow and oxygenation enough to initiate a cascade of neuronal injuries that continues for hours to days.^12^, ^13^, ^14^, ^15^ TH reduces cellular metabolism to blunt this cascade. If applied rapidly, within a 6-h therapeutic window, TH limits injuries and prevents delayed cerebral energy failure. HIE treatment decisions are typically guided by neurological exam and electroencephalography (EEG), with outcome assessed by post-TH MRI. However, these approaches are subjective, nonspecific, and have had limited prognostic value after the introduction of TH.^16^^,^^17^ Research is ongoing to improve diagnostics for HIE in the era of TH, for example by quantitative analysis of EEG.^18^

Because cerebral metabolism is directly related to the pathophysiology of HIE and the therapeutic effect of TH, measuring the cerebral metabolic rate of oxygen consumption (CMRO_2_) has enormous potential to improve diagnostic and prognostic accuracy in neonatal encephalopathy.^19^ Non-invasive measurement of cortical CMRO_2_ recently became feasible at the bedside in neonatal intensive care units (NICUs) through a combination of frequency-domain near-infrared and diffuse correlation spectroscopies (FDNIRS-DCS).^20^, ^21^, ^22^ We previously showed that CMRO_2_ was reduced during TH and increased upon rewarming to normothermia, demonstrating CMRO_2_ is sensitive to effects of treatment with TH.^21^ If TH is successful, more neurons and synapses would survive the hypoxic-ischemic insult and subsequent cascade, resulting in higher CMRO_2_ after treatment and likely improved neurodevelopmental outcomes. This study aimed to test the hypothesis that CMRO_2_ following TH in neonatal HIE predicts neurodevelopmental outcomes at 18 months of age.

## Methods

### Study participants

In this prospective, multicentre, observational, cohort study, participants were recruited from the NICUs of Boston Children's Hospital (BCH), Brigham and Women's Hospital (BWH), and Beth Israel Deaconess Medical Center (BIDMC) between December 2015 and October 2019 and followed through July 2021.

Medical records were screened for eligibility upon NICU admission. The inclusion and exclusion criteria followed clinical guidelines for eligibility for TH for perinatal distress/asphyxia and neonatal encephalopathy.^23^ Per the guidelines, TH was indicated for neonates aged 36 weeks gestational age (GA) or older and less than 6 h old, and considered at the discretion of the care team for those aged 34 weeks GA or older and less than 12 h old. The study included all neonates meeting the clinical eligibility criteria for TH, regardless of sex, with enrolment up to 72 h after birth. Exclusion criteria were less than 34 weeks GA, older than 72 h, presence of major congenital anomalies, severe intrauterine growth restriction, major intracranial haemorrhage, systemic or central nervous system infection, significant coagulopathy, or TH not initiated.

### Ethics

This study was approved by the institutional review board (IRB) of BCH (Ref: IRB-P00012473) with BCH as the reviewing institution and BWH and BIDMC as relying institutions. This study is registered at ClinicalTrials.gov with the identifier NCT02793999. All patients’ parents or guardians provided signed informed consent.

### TH protocol

TH was performed in three phases according to clinical protocols: 1) the cooled (C) phase, controlled hypothermia for 72 h with a goal temperature of 33.5 °C typically initiated within 6 h of birth but considered starting at up to 12 h of age; 2) the rewarming (RW) phase, during which neonates were slowly returned to normothermia over 12 (BCH and BIDMC) or 15 (BWH) hours at a controlled, constant rate; and 3) the normothermic (NT) phase. Sedation during C was maintained primarily with morphine. Anti-seizure medications, typically phenobarbital, were used as clinically indicated. Study measurements were categorised by TH phase (C, RW, or NT) according to designations in the chart.

### Clinical and demographic data

Clinical, demographic, and core temperature variables were collected from medical records. Pulse oximetry values (SpO_2_) were obtained from the patient monitor during FDNIRS-DCS measurements. Median family income for participants' block group in the U.S. Census Bureau's American Community Survey (ACS) 5-year estimate for 2014–2019 was a proxy for socioeconomic status (SES).^24^

### Neonatal encephalopathy (NE) score

NE scores were determined with the BCH encephalopathy scoring system which ranges from 0 to 16 with higher scores indicating worse encephalopathy (Supplement Table S1). The BCH system was derived from the Sarnat score; it is a simplified version of the Sarnat score that includes high yield items from the Sarnat exam derived from analysis of Sarnat data. Severity of HIE was categorised as mild (NE score <4), moderate (4–10) or severe (>10). If a patient received multiple exams, the worst of early NE scores was used. NE scoring was blinded to research results and neurodevelopmental outcomes.

### MRI categorization of brain injuries

Clinical brain magnetic resonance imaging (MRI) was typically performed on day four. MRI studies included volumetric T1 weighted, axial T2 weighted and diffusion weighted imaging as well as MR spectroscopy (MRS) of the left basal ganglia/thalamus with TE = 35 ms and 135 ms. MRI images were scored using the National Institute of Child Health and Human Development (NICHD) Neonatal Research Network (NRN) score^25^ by an experienced paediatric neuroradiologist (PEG) while blinded to clinical history and research results. The reviewer also qualitatively assessed MRS for evidence of lactate by the presence of a short echo doublet at 1.33 ppm that inverted on long echo. A second set of scores and evidence of lactate were generated from each scan's associated contemporaneous clinical radiology report independently of the neuroradiologist's scores from the images and spectra. In cases in which the scores disagreed, another radiologist (EY) blindly scored the MRI and MRS data and the final score was the majority consensus of the three scores.

### Cerebral metabolic rate of oxygen consumption (CMRO_2_) measurements

CMRO_2_ was determined from measurement of cerebral tissue haemoglobin oxygen saturation (ctSO_2_) and cerebral blood flow index values (CBF_i_) by FDNIRS-DCS (MetaOx, ISS Inc.).^20^, ^21^, ^22^ Measurements were performed daily using identical procedures at each site, starting as soon as feasible after enrolment and continuing until discharge or six days after birth. Measurements were unscheduled but occurred while the neonate was quietly resting, avoiding clinical care or stimulation. An FDNIRS-DCS probe was held for 30 s on left and right frontal locations on the forehead and repeated three times at each location. Replicates from both sides were averaged together for analysis. FDNIRS-DCS data were analysed offline with clinical staff blinded to measurements and researchers blinded to clinical assessments and outcomes. Cerebral fractional tissue oxygen extraction (cFTOE) was calculated by $cFTOE=\left({SpO}_{2}-{ctSO}_{2}\right)/{SpO}_{2}$. CMRO_2_ index (CMRO_2i_) was calculated by ${CMRO}_{2i}=HGB\cdot {CBF}_{i}\cdot \left({SpO}_{2}-{ctSO}_{2}\right)\text{,}$ where haemoglobin level (*HGB*) was determined from table values by postnatal age at measurement.^26^

### Outcome measurements

Follow-up was performed at 18 months by a trained administrator to determine cognitive, language, and motor development using the Bayley Scales of Infant and Toddler Development, Third Edition (BSID-III). Administrators were blinded to both patient's clinical history and FDNIRS-DCS results.

### Statistics

To compare demographic and clinical characteristics between two groups of patients we used tests appropriate to the distribution of each variable (binary or categorical, continuous, ordinal, skewed). Fisher Exact test was used for binary or categorical variables. Student 2-tailed, unpaired t-test was used for continuous variables. Normality of each continuous variable was evaluated by the Shapiro–Wilk test. Mann–Whitney U test was used for ordinal variables or continuous variables with skewed distribution according to the Shapiro–Wilk test. To analyse NIRS measures and BSID-III scores we employed mixed linear models. Discrete fixed effects included TH phase, site (and alternatively as a random effect), sedative use, phenobarbital use, brain injury, and follow-up subgroup. Continuous fixed effects included temperature, NE score, NIRS measures (with BSID-III as outcome), and neighbourhood family income. Sex was considered as an effect modifier to the regression model. For NIRS measures taken repeatedly per patient, we included a random effect to account for within-patient correlation. Given the narrow temperature range, a linear model was used for temperature effects on haemodynamics. We tested for possible bias in missing baseline data by comparing demographic and clinical characteristics of participants excluded because of poor signal quality with those of the included sample. We tested for attrition bias in the neurodevelopmental assessments by comparing demographic and clinical characteristics, as well as the haemodynamic variables at each TH phase, between participants with 18-month data and those lost to follow-up. *P* < 0.05 was the criterion for statistical significance in all analyses. No adjustment was made for multiple comparisons, as justified for three-group comparisons by the principle of closed testing^27^ and otherwise by allocating a 5% type I error rate to each specific physiological and developmental hypotheses.^28^ SAS software (version 9.4) was employed for all statistical computations. Data collection and analysis was generally blinded as described in the relevant methods subsections. Sample size was determined by the available case load and consent rate during the study period.

### Role of funder

The funder of the study had no role in study design, data collection, analysis, interpretation, or writing the manuscript or the decision to submit it for publication.

## Results

### Study sample

**Fig. 1:**
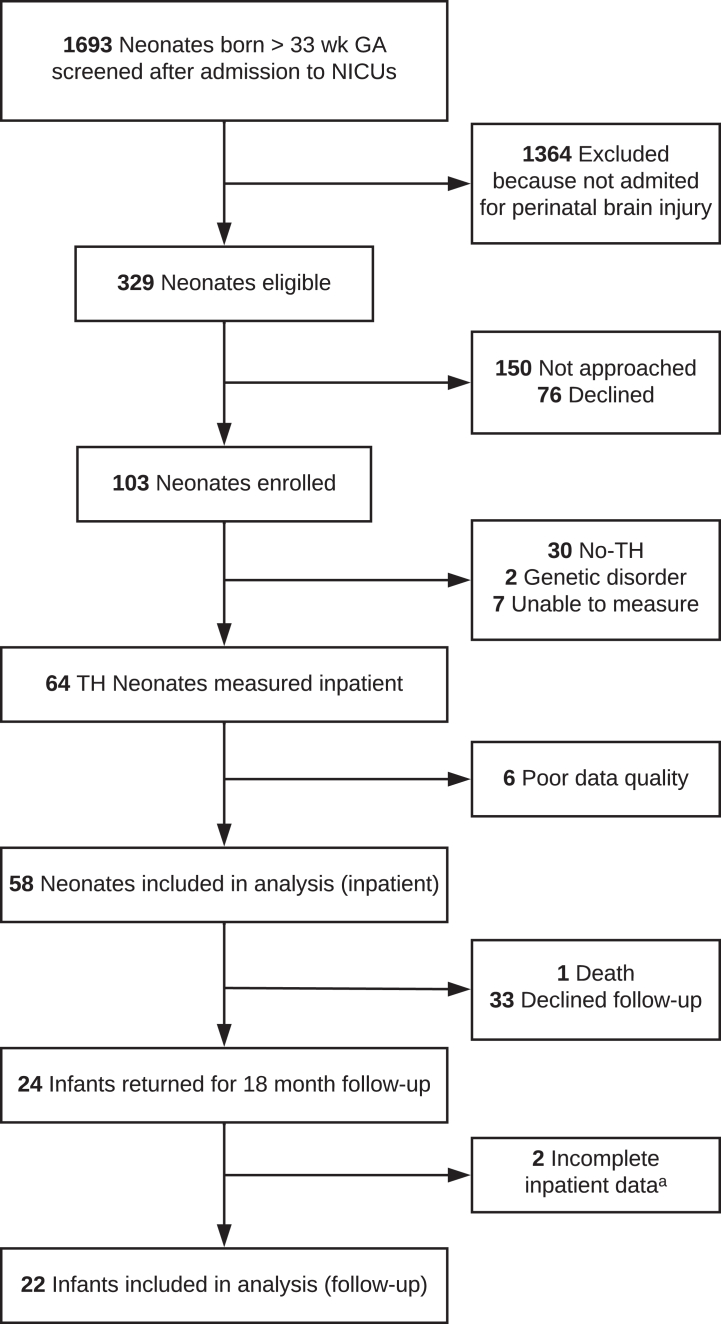
**Study cohort flow diagram.** Abbreviations: GA, Gestational age; TH, Therapeutic hypothermia. ^a^ Two incomplete due to no NT data.

During recruitment, we screened 1693 admissions meeting the age criteria, with 329 neonates eligible for recruitment based on clinical consideration for TH. One hundred fifty were not approached for logistical reasons or by judgement of medical staff. Among the 173 approached, 103 enrolled, of which 39 were excluded due to clinical decisions not to proceed with TH, genetic disorders, or inability to obtain a measurement, leaving 64 neonates with HIE undergoing TH measured (Fig. 1).

With six participants FDNIRS-DCS signal quality was too poor to estimate cerebral haemodynamic values, leaving 58 neonates included in the analysis. One died before discharge from complications unrelated to HIE. Twenty-four infants returned for follow-up assessments with the BSID-III. Two were without NT FDNIRS-DCS data, leaving 22 infants (mean postnatal age (SD) 19.1 (1.2) months) included in the outcome analysis.

Baseline characteristics of the participants (n = 58) are listed in Table 1. Ninety-five percent (55) were from Massachusetts, with the remainder from surrounding states. Median census block family income (mean ± SE $118.4 ± 7.0 k) was greater than that of Massachusetts ($103.1 ± 0.4 k, *P* = 0.03; Z-test) and the United States ($77.3 k ± 0.1 k, *P* < 0.001; Z-test). Overall, HIE was mild to moderate with 10-min Apgar score median (IQR) = 7 (6–8) and worst NE score median (IQR) = 4 (3–7). Additionally, 67% (38) had no evidence of brain injury on clinical MRIs. Most were cooled within 6 h after birth, with four within 6–6.5 h and three within 6.5–12 h. The three patients actively cooled after 6.5 h were transport patients from community hospitals, with two having documentation of passive cooling starting by around 6 h. Two patients were rewarmed early because of improvement within 19–24 h of TH. All these participants were included in the analysis, because all met the TH protocol's criteria to allow these changes at the discretion of treating physicians. Sixty-eight percent (39) had records of receiving early intervention services after discharge. Baseline characteristics of the subgroups with and without 18-month outcomes did not differ significantly (Table 1), except for lower recorded umbilical cord blood pH in the outcome group (mean ± SE 6.98 ± 0.02 vs 7.05 ± 0.03, *P* = 0.04; Student 2-tailed unpaired t-test). Baseline characteristics of included participants did not differ significantly from excluded participants (Supplement Table S2).

**Table 1:** Baseline maternal and infant characteristics of the entire exposure group and by follow-up subgroup.

Variable	All TH patients (n = 58)	TH patients without 18-month outcome (n = 36)	TH patients with 18-month outcome (n = 22)	*P* value^a^
**Maternal characteristics**				
Age, mean (SD), y	32.0 (5.7)	31.6 (6.3)	32.6 (4.4)	0.50^b^
Family income, $1,000^e^, mean (SD)	118.4 (53.5)	118.3 (54.9)	118.5 (52.3)	0.99^b^
**Infant characteristics**				
Gestational age at birth, mean (SD), wk	39.3 (1.5)	39.1 (1.7)	39.6 (1.2)	0.28^b^
Girls, No. (%)	22 (38)	11 (31)	11 (50)	0.17^c^
Birth weight, mean (SD), g	3293 (624)	3203 (637)	3442 (588)	0.16^b^
1-min Apgar score, median (IQR)	2 (1–4)	2 (1–4)	3 (1–5)	0.44^d^
5-min Apgar score, median (IQR)	6 (4–7)	6 (3–8)	6 (5–7)	0.66^d^
10-min Apgar score, median (IQR)	7 (6–8)	7 (5–7)	7 (6–8)	0.68^d^
Lowest recorded umbilical pH^f^, mean (SD)	7.02 (0.13)	7.05 (0.15)	6.98 (0.11)	0.04^b^
Lowest recorded postnatal pH^g^, mean (SD)	7.17 (0.13)	7.16 (0.12)	7.18 (0.14)	0.52^b^
Worst NE Score, median (IQR)	4 (3–7)	5 (3–7)	4 (3–6)	0.20^d^
Length of hospital stay, median (IQR), d	6 (6–12)	6 (6–12)	7 (6–12)	0.90^d^
Patients with seizures during NICU stay, No. (%)	10 (17)	6 (17)	4 (18)	1.00^c^
Patients intubated during NICU stay, No. (%)	19 (33)	15 (42)	4 (18)	0.09^c^
Patients rewarmed early from TH, No. (%)	2 (4)	1 (3)	1 (5)	1.00^c^
Normothermic MRI categorization^h^				
No Injury, No. (%)	38 (67)	22 (63)	16 (73)	0.57^c^
Evidence of Injury, No. (%)	19 (33)	13 (37)	6 (27)	0.57^c^
Death before age 2 y, No. (%)	1 (2)	1 (3)	0 (0)	1.00^c^
Followed by early intervention services, No. (%)	39 (68)	22 (63)	17 (77)	0.38^c^
Age at BSID-III follow-up, mean (SD), m	–	–	19.1 (1.2)	

Abbreviations: BSID-III, Bayley Scales of Infant and Toddler Development, Third Edition; IQR, Interquartile range; MRI, Magnetic resonance imaging; NICU, Neonatal intensive care unit; NE, Neonatal encephalopathy; TH, Therapeutic hypothermia.

a Comparing subgroups with and without 18-month outcome.

b *P* values by Student 2-tailed, unpaired t-test.

c *P* values by Fisher Exact test.

d *P* values by Mann–Whitney U test.

e Obtained from US Census data.

f Umbilical cord blood pH missing for five neonates.

g Blood pH within 4 h of birth; missing for one neonate.

h One patient did not receive a clinical MRI; hence n = 57.

### Association between cerebral haemodynamics and temperature

Two hundred twenty-one FDNIRS-DCS measurements were collected from 58 neonates, with 72, 70, and 79 measurements during the C, RW and NT phases of TH, respectively. Median (IQR) postnatal ages of the neonates during measurements were 50 (37–64) hours, 82 (79–86) hours, and 117 (102–137) hours for the C, RW, and NT phases, respectively. Mean (SD) patient temperatures during measurements were 33.5 (0.3) °C, 34.9 (0.7) °C, and 36.8 (0.5) °C for the C, RW, and NT phases, respectively.

**Fig. 2:**
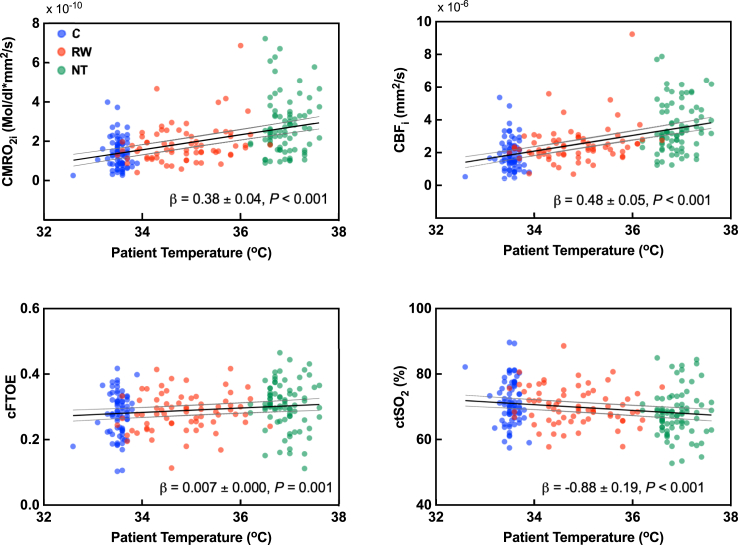
**Association of cerebral haemodynamic variables with body temperature**. Lines indicate fitted values from mixed linear model with random intercept for patients and 95% confidence limits (n = 221 observations from 58 patients). *P* tests H_0_: β = 0. Abbreviations: C, Cooled; CBF_i_, Index of cerebral blood flow; cFTOE, Cerebral fractional tissue oxygen extraction; CMRO_2i_, Index of cerebral metabolic rate of oxygen consumption; ctSO_2_, Cerebral tissue oxygen saturation; NT, Normothermic; RW, Rewarming; β, Regression coefficient ± standard error (SE), in units of cerebral haemodynamic variable per °C.

When compared by TH phase, CMRO_2_ and CBF were lowest during C and increased significantly in RW and NT. cFTOE was highest at NT with ctSO_2_ correspondingly lowest (Supplement Figure S1 and Supplement Table S3). Haemodynamic variables changed linearly with temperature (Fig. 2) with rates relative to NT of 14.4% (95% CI, 14.2–14.6), 14.0% (95% CI, 13.7–14.2), 2.2% (95% CI, 2.1–2.4) and −1.3% (95% CI, −1.3 to −1.2) per °C, for CMRO_2i_, CBF_i,_ cFTOE and ctSO_2,_ respectively. A multivariable analysis was performed controlling for site, sedative use, and anti-seizure medication, but associations did not change (Supplement Table S4). Excluding the five participants with active TH starting later than 6.5 h or early rewarming had negligible impact on the regression results.

### Clinical MRI and MRS

Clinical MRI studies were obtained in 57 of 58 neonates on median of day 3.9 (IQR, 3.4–4.4). No MRI was ordered for one neonate rewarmed early. Thirty-eight (67%) scored normal, 8 (14%) scored 1a, 1 (2%) scored 1 b, 6 (7%) scored 2a, 5 (9%) scored 2 b and 1 (2%) scored 3. MRS was poor quality in six and not acquired in two. In the remaining 49, lactate was present in 3 (6%) and absent in 46 (94%).

### Association between cerebral haemodynamics, NE score and MRI

None of the haemodynamic variables at any phase of TH were associated with NE score (Supplement Figure S2 and Supplement Table S5). Haemodynamic group averages were not significantly different between those with and without MRI evidence of injury (Supplement Table S6). Also, the NE scores of these two MRI groups did not differ (*P* = 0.95; Student 2-tailed unpaired t-test), even when grouped by those with and without 18-month outcomes (*P* = 0.50 and 0.62, respectively; Student 2-tailed unpaired t-test).

### Neurodevelopmental outcome

Mean (SD) BSID-III composite scores for the 22 infants in the subgroup with 18-month outcomes were 106.4 (11.9), 104.4 (10.7), 102.8 (7.6) in the cognitive, language, and motor domains, respectively. No significant differences in group averages of cerebral haemodynamic variables for TH phases were observed between subgroups with and without 18-month outcomes (Supplement Table S7).

### Association between neonatal data and neurodevelopmental outcome

**Fig. 3:**
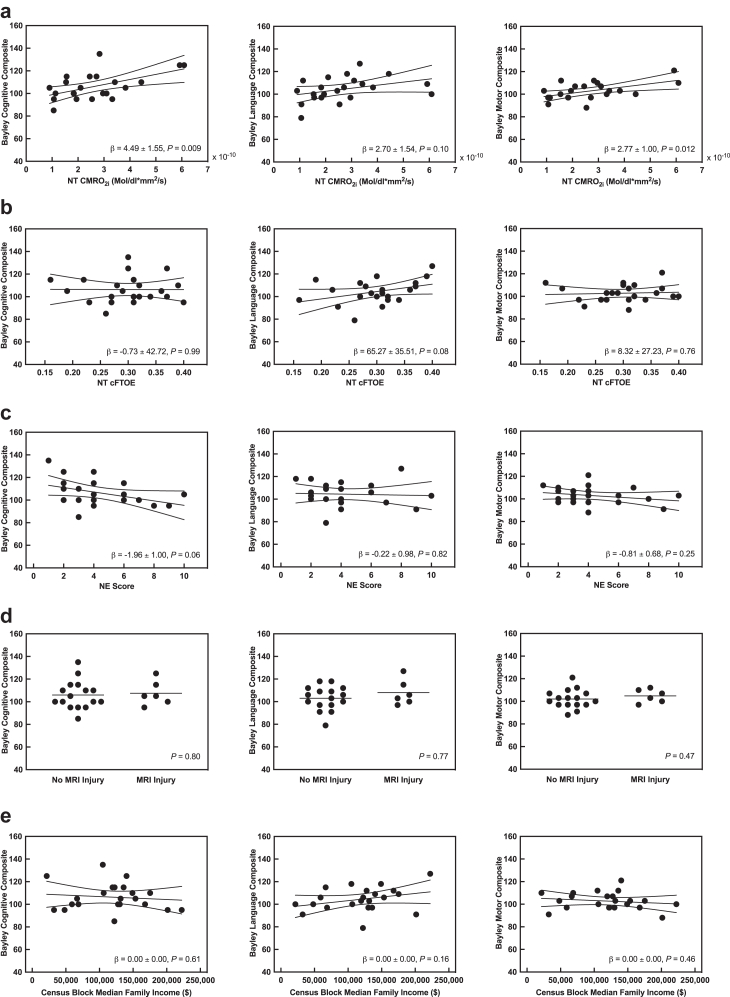
**Bayley scales of infant and toddler development, third edition composite scores in children surviving hypoxic ischemic encephalopathy: association of neonatal variables with cerebral haemodynamic measures at normothermia following therapeutic hypothermia (a, b), neonatal variables (c, d), and socioeconomic status (e).** Lines in panels a, b, c and e indicate fitted linear regression model and 95% confidence limits (n = 22). *P* tests H_0_: β = 0. Lines in panel d indicate group means (two-sided Student's *t*-test, n = 22). Abbreviations: cFTOE, Cerebral fractional tissue oxygen extraction; CMRO_2i_, Index of cerebral metabolic rate of oxygen consumption; MRI, Magnetic resonance imaging; NE, neonatal encephalopathy; NT, Normothermia; β, Regression coefficient ± standard error, in Bayley scale points per unit change in explanatory variable.

CMRO_2_ in NT was associated positively with BSID-III cognitive and motor composite scores at 18 months (β (SE), 4.49 (1.55) BSID-III points per 10^−10^ mol/dL × mm^2^/s; R^2^ = 0.30; *P* = 0.009; and β (SE), 2.77 (1.00) BSID-III points per 10^−10^ mol/dL × mm^2^/s; R^2^ = 0.28; *P* = 0.01; respectively; linear regression; Fig. 3a). No association was observed in the language domain. CBF in NT was also associated positively with cognitive and motor scores (β (SE), 4.59 (1.28) BSID-III points per 10^−6^ mm^2^/s; R^2^ = 0.39; *P* = 0.002 and β (SE), 2.62 (0.87) BSID-III points per 10^−6^ mm^2^/s; R^2^ = 0.31; *P* = 0.007; respectively; linear regression; Supplement Table S8), while neither cFTOE nor ctSO_2_ showed any associations (Fig. 3b, Supplement Table S8). NE score had a moderate effect size on BSID-III cognitive composite scores (β (SE), −1.96 (1.00) BSID-III points per unit change in NE; R^2^ = 0.16; *P* = 0.06; linear regression; Fig. 3c). Subgroups with and without MRI evidence of brain injury had no significant differences between the BSID-III composite scores (Fig. 3d). Census income was not associated with BSID-III scores (Fig. 3e). Because both CMRO_2_ and NE score had a moderate effect size on BSID-III cognitive composite scores, a multivariable regression was performed between haemodynamic variables in NT and BSID-III outcomes when controlling for NE score, but changes in effect and significance were negligible (Supplement Table S9). Similarly, since temperature had a moderate effect on CMRO_2_, a multivariable analysis was performed controlling for temperature, but associations did not change (Supplement Table S9). No sedatives were used at NT and associations did not change when controlled for phenobarbital use (Supplement Table S9). We tested for sex as an effect modifier and found no difference between females and males (Supplement Table S10). Excluding the one participant with active TH starting later than 6.5 h and the one rewarmed early had negligible impact on the results. There were no associations between haemodynamic variables in C or RW and BSID-III composite scores (Supplement Table S8).

## Discussion

We prospectively measured cerebral haemodynamics by FDNIRS-DCS in neonates receiving TH for HIE and found CMRO_2_ and CBF were the only neonatal predictors of neurodevelopmental outcomes at 18 months. Because the clinical severity of HIE in our cohort was mild to moderate, BSID-III outcomes spanned a normal range with greater CMRO_2_ after rewarming associated with better cognitive and motor scores. No association with outcome was found with NE score, SES, MRI injury score, or cFTOE. CMRO_2_ uniquely predicted cognitive and motor outcomes above and beyond current clinical standards.

The relationship between cerebral metabolism and neurodevelopmental outcome in neonatal HIE treated with TH has previously been investigated by MRS. MRS typically assesses cerebral metabolism by measurements of metabolites such as lactate, N-acetylaspartate (NAA) or their ratio. Quantitative measurements of metabolites after TH by research MRS were recently shown to be associated with severe outcomes defined as death or BSID-III composite score <85 at 2 years.^29^, ^30^, ^31^ However, the overall predictive power of these MRS measures is controversial,^32^ especially because the association is lost as BSID-III scores approach normal after mild to moderate HIE.^33^ Although we did not perform quantitative research MRS, we found qualitative evidence of lactate in only 6% of clinical scans. CMRO_2_ and MRS measure different aspects of cerebral metabolism with FDNIRS-DCS CMRO_2_ monitoring instantaneous metabolic activity in the cortex, whereas MRS represents cumulative metabolic stress over longer durations, typically in deep brain structures. This distinction may be responsible for the greater sensitivity of CMRO_2_ to predict outcomes after TH in mild to moderate HIE in the current study.

With the advent of TH, the primary role of the NE score has become guiding decisions on whom to cool, typically within the 6-h window, but remain highly subjective.^10^^,^^34^ In our cohort, association between NE and BSID-III cognitive scores did not reach significance, but had a modest effect size. Controlling for NE score slightly reduced the effect size of NT CMRO_2_ without affecting significance, indicating the NE score had a minor, independent contribution to outcomes. While SES may be expected to affect outcomes,^2^^,^^35^ we did not observe an association in our relatively homogeneous demographic sample.

Structural and diffusion MRI are clinical standards for evaluating brain injuries associated with HIE, with sensitivity peaking days to weeks after treatment.^36^^,^^37^ In fact, neonatal MRI after completion of TH is the principal tool used by clinicians and researchers to predict neurodevelopmental outcomes.^25^^,^^38^ However, in the era of TH, injuries are only reported in about half of cases.^39^ In our cohort, 67% of participants had no evidence of injury on MRI and MRI score had no association with either BSID-III scores or CMRO_2_. Previous MRI studies evaluated predictions against severe categorical outcomes, but we showed a dose-dependent linear relationship between CMRO_2_ and outcome, sensitive to mild HIE. Thus, our data demonstrate that in mild-to-moderate HIE treated with TH, CMRO_2_ has better prognostic power than currently used MRI measures.

Measurements of cFTOE or ctSO_2_ during TH with clinical NIRS oximeters have been investigated previously for association with later neurodevelopmental outcomes. However, results have been too few and inconsistent to establish evidence-based guidelines.^40^^,^^41^ Consistent with prior studies,^42^, ^43^, ^44^, ^45^, ^46^, ^47^ we found no association between cFTOE or ctSO_2_ and mild to moderate outcomes. Furthermore, net changes in CMRO_2_ and CBF from cooled to normothermia were consistent with neurovascular coupling and about tenfold greater than for cFTOE and ctSO_2_. The difference in sensitivity of these variables reflects the active physiological regulation of CBF vs the tendency of cFTOE for homeostasis.^48^ ctSO_2_ is often colloquially considered an indicator of perfusion, but as can be seen from the Fick principle and conservation of mass, cFTOE and ctSO_2_ are proportional to CBF only when CMRO_2_ is constant. Our results are real-world example of this subtle point and how this simplification breaks down under clinically important conditions. Although results with CMRO_2_ and CBF were similar in this study, CBF, like cFTOE and ctSO_2_, can be affected by causes unrelated to brain activity, such as hypercapnia. In contrast, CMRO_2_ is brain specific because changes in CBF and cFTOE from non-brain effects tend to cancel out according to the Fick principle. In fact, one of the earliest results from the development of radioactive CBF tracers was the demonstration that CMRO_2_ does not change with arterial CO_2_ level.^49^ Our results are consistent with our hypothesis and show CMRO_2_ is fundamentally a better physiological indicator of TH treatment effects and outcomes.

This study also has limitations. Although our study did not intentionally target mild HIE, our cohort were majority mild to moderate cases. Fewer families likely returned for follow-up because of the mild HIE. However, differences in baseline variables and exposures in the groups with and without outcome were minimal and statistically insignificant, except for an isolated finding of a group difference in umbilical cord pH. We do not place much importance in this difference because umbilical cord pH was not always reported in the clinical record and both groups were highly acidotic, below the 7.10 threshold for consideration for TH.^23^^,^^50^ Our cohort was wealthier than average, but we did not discriminate enrolment by wealth and is representative for high income countries. However, the hypothermia for moderate or severe neonatal encephalopathy in low-income and middle-income countries (HELIX) randomised controlled trial questioned the efficacy of TH for HIE in low- and middle-income countries (LMICs), possibly because of differences in the aetiology of the disease.^51^ Studies in LMICs are needed to investigate relationships between HIE and CMRO_2_ in low resource settings. We also acknowledge the sample size was small, but associations between exposure and outcomes had robust statistical significance, even after controlling for potential confounding factors. Additional research is necessary to prove the superior role of FDNIRS-DCS-derived CMRO_2_ with respect to neurodevelopmental outcome.

In conclusion, our results suggest that higher CMRO_2_ just after TH predicts better neurodevelopmental outcome, introducing a potential objective, quantitative measure to the limited number of prognostic indicators for mild and moderate HIE. In fact, treatment for mild HIE is a critical question that has been understudied.^9^^,^^10^^,^^52^ Finder et al. recently found outcomes in mild untreated HIE, while within normal range, were lower than controls.^8^ In this study, CMRO_2_ was able to predict outcomes, even in mild HIE, making it potentially useful as a short-term endpoint in future neuroprotection trials. Finally, we have shown CMRO_2_ is sensitive to hypothermia treatment and can be measured repeatedly at point of care. Future work will investigate continuous monitoring of CMRO_2_ as a diagnostic from the earliest suspicion of HIE with the goal of developing actionable information to optimise individual NICU care and outcome.

## Contributors

All authors read and approved the final version of the manuscript. Concept and design: Sutin, Lin, Grant. Acquisition, analysis, or interpretation of data: All authors. Drafting of the manuscript: Sutin, Vyas, Feldman, Lin, Grant. Critical revision of the manuscript for important intellectual content: Sutin, Feldman, Soul, Lin, Grant. Statistical analysis: Vyas, Feldman, Lin. Obtained funding: Grant. Administrative, technical, or material support: All authors. Supervision: Lin, Grant. Drs Sutin, Lin, and Grant had full access to all the data in the study and takes responsibility for the integrity of the data and the accuracy of the data analysis.

## Data sharing statement

De-identified data can be made available from the corresponding author JS upon reasonable request. Contact information for JS is included on the title page.

## Declaration of interests

The authors declare no conflict of interest.
