# Supplementary material for: Association of cerebral metabolic rate following therapeutic hypothermia with 18-month neurodevelopmental outcomes after neonatal hypoxic ischemic encephalopathy
Source: eBioMedicine. 2023 Jun 29;94:104673. doi: 10.1016/j.ebiom.2023.104673 (PMC10338207; doi:10.1016/j.ebiom.2023.104673)
Supplement: Supplement Figures S1–S2 and Tables S1–S10 [file mmc1.docx]

**Supplementary Information**

**Supplementary Table 1.** Boston Children’s Hospital Neonatal Encephalopathy (NE) Scale

**Supplementary Table 2.** Baseline Maternal and Infant Characteristics of the Entire Exposure Group and Patients Excluded from Analysis

**Supplementary Figure 1.** Cerebral Haemodynamic Variables by Therapeutic Hypothermia Phase

**Supplementary Table 3.** Tests of Group Differences in Cerebral Haemodynamic Variables by Therapeutic Hypothermia Phase

**Supplementary Figure 2.** Association Between Neonatal Encephalopathy Score and Normothermic Cerebral Haemodynamic Variables

**Supplementary Table 4.** Association of Cerebral Haemodynamic Variables with Temperature with Site, Sedatives, and Phenobarbital as Covariates

**Supplementary Table 5:** Association between Neonatal Encephalopathy Score and Cerebral Haemodynamic Variables

**Supplementary Table 6.** Test of Group Differences in Cerebral Haemodynamic Variables Between MRI Injury Subgroups at Each Therapeutic Hypothermia Phase

**Supplementary Table 7.** Test of Group Difference in Cerebral Haemodynamic Variables Between Follow-up Subgroups at Each Therapeutic Hypothermia Phase

**Supplementary Table 8.** Association of Neonatal Variables with BSID-III Composite Scores

**Supplementary Table 9.** Association of Cerebral Haemodynamic Variables at Normothermia with BSID-III Composite Scores with Temperature, Neonatal Encephalopathy Scores, and Phenobarbital as Covariates

**Supplementary Table 10.** Sex as an Effect Modifier to the Association of Cerebral Haemodynamic Variables at Normothermia with BSID-III Composite Scores

**Supplementary Table 1. Boston Children’s Hospital Neonatal Encephalopathy (NE) Scale**

| **Sign** | **0** | **1** | **2** | **3** | **4** |
| --- | --- | --- | --- | --- | --- |
| **Level of Consciousness** | Normal |  | Hyperalert/  Irritable or sleepy | Lethargic or suspected clinical seizures | Stuporous/  Comatose |
| **Spontaneous Activity** | Normal |  | Decreased | Absent |  |
| **Muscle Tone/Posture** | Normal | Hypertonic | Hypotonic | Flaccid |  |
| **Moro Reflexes** | Normal | Exaggerated | Weak/Incomplete | Absent |  |
| **Respirations** | Normal |  | Periodic Breathing | Apnoea |  |

NE score is the total of the sign scores.

**Supplementary Table 2. Baseline Maternal and Infant Characteristics of the Entire Exposure Group and Patients Excluded from Analysis**

| **Variable** | **All TH Patients Included in Inpatient Analysis**  **(n = 58)** | **TH Patients Excluded  from Analysis  (n = 6)** | ***P* value^a^** |
| --- | --- | --- | --- |
| Age, median (IQR), y | 32·5 (28·0-36·0) | 31·0 (29·0-33·0) | 0·36^b^ |
| Family income, $1,000^d^, median (IQR) | 122·3 (68·8-145·9) | 147·4 (110·9-167·7) | 0·28^b^ |
| Gestational age at birth, median (IQR), wk | 39·4 (38·6-40·6) | 39·6 (38·0-40·3) | 0·91^b^ |
| Girls, No. (%) | 22 (38) | 1 (17) | 0·41^c^ |
| Birth weight, median (IQR), g | 3210 (2870-3773) | 3260 (2910-3395) | 0·74^b^ |
| 1-min Apgar score, median (IQR) | 2 (1-4) | 2 (1-3) | 0·65^b^ |
| 5-min Apgar score, median (IQR) | 6 (4-7) | 6 (4-6) | 0·60^b^ |
| 10-min Apgar score, median (IQR) | 7 (6-8) | 7 (6-9) | 0·68^b^ |
| Lowest recorded umbilical pH^e^, median (IQR) | 7·01 (6·93-7·10) | 6·93 (6·88-7·11) | 0·45^b^ |
| Lowest recorded postnatal pH^f^, median (IQR) | 7·17 (7·10-7·27) | 7·13 (7·04-7·18) | 0·41^b^ |
| Worst NE Score, median (IQR) | 4 (3-7) | 6 (5-9) | 0·30^b^ |
| Length of hospital stay, median (IQR), d | 6 (6-12) | 5 (4-7) | 0·05^b^ |
| Patients with seizures during NICU stay, No. (%) | 10 (17) | 2 (33) | 0·31^c^ |
| Patients intubated during NICU stay, No. (%) | 19 (33) | 2 (33) | 1·00^c^ |
| Patients rewarmed early from TH, No. (%) | 2 (4) | 0 (0) | 1·00^c^ |
| Normothermic MRI categorisation^g^ |  |  |  |
| No Injury, No. (%) | 38 (63) | 2 (40) | 0·34^c^ |
| Evidence of Injury, No. (%) | 19 (37) | 3 (60) | 0·34^c^ |
| Death before age 2 y, No. (%) | 1 (2) | 0 (0) | 1·00^c^ |
| Followed by early intervention services, No. (%) | 39 (68) | 5 (83) | 0·66^c^ |

Abbreviations: IQR, interquartile range; MRI, magnetic resonance imaging; NE, neonatal encephalopathy; NICU, neonatal intensive care unit; TH, therapeutic hypothermia.

^a^ Comparing subgroups included in and excluded from analysis. ^b^ *P* values by Mann-Whitney U test. ^c^ *P* values by Fisher Exact test. ^d^ Obtained from US Census data. ^e^ Umbilical cord blood pH missing for five neonates. ^f^ Blood pH within four hours of birth; missing for one neonate. ^g^ One patient did not receive a clinical MRI; hence n = 57.

**
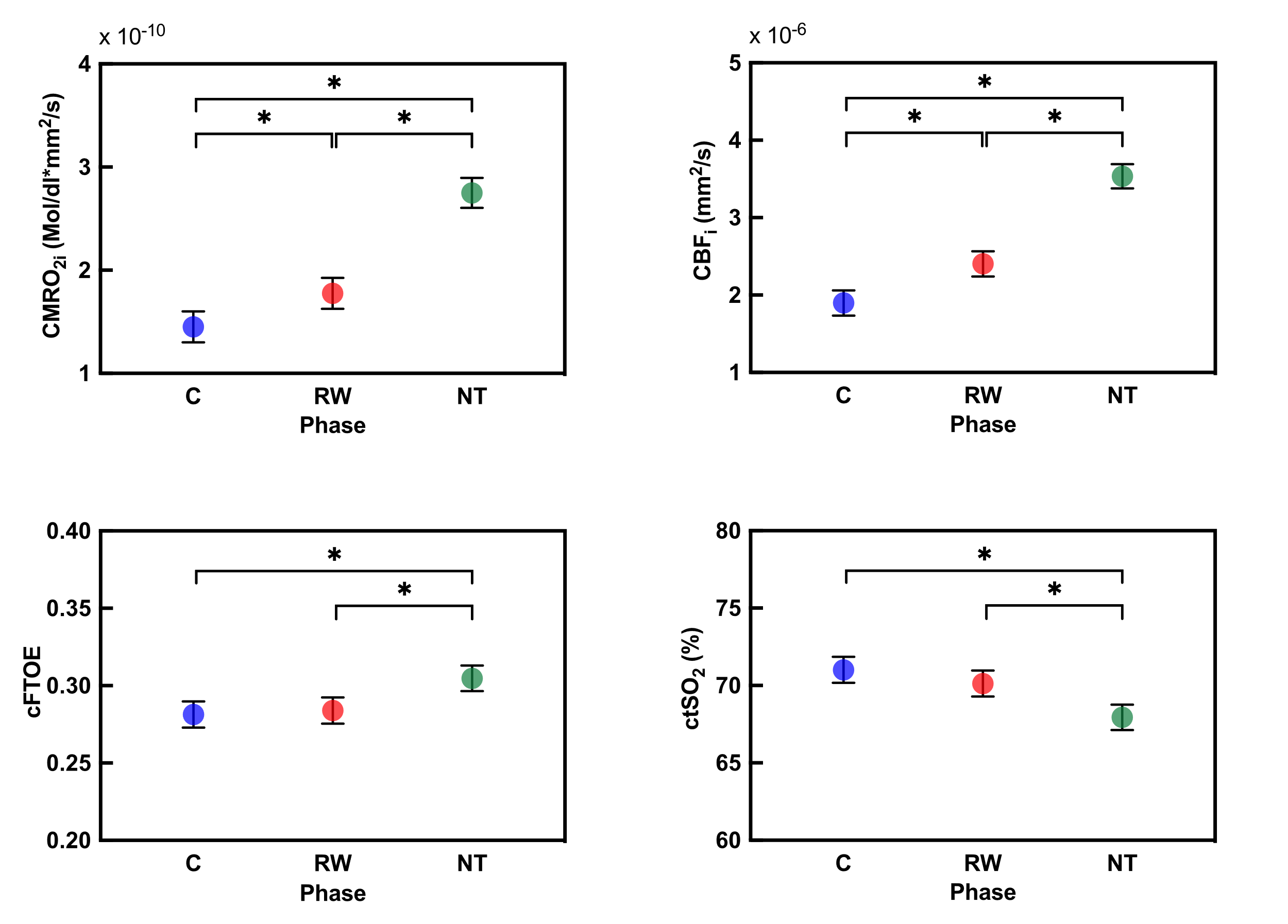
**

**Supplementary Figure 1. Cerebral Haemodynamic Variables by Therapeutic Hypothermia Phase**

Dots indicate estimated mean values and error bars indicate standard errors from the linear mixed-effects model. Blue, red and green dots indicated the cooled, rewarming and normothermic phase. Statistical significance of P < 0·05 accounting for within-patient correlation within and across phases is denoted by * between two phases.

Abbreviations: C, cooled; CBF_i_, index of cerebral blood flow; cFTOE, cerebral fractional tissue oxygen extraction; CMRO_2i_, index of cerebral metabolic rate of oxygen consumption; ctSO_2_, cerebral tissue oxygen saturation; NT, normothermic; RW, rewarming.

**Supplementary Table 3. Tests of Group Differences in Cerebral Haemodynamic Variables by Therapeutic Hypothermia Phase**

| **Haemodynamic Variables** | **Overall *P* value** | **Pairwise Comparison Between Phases** | | | |  |
| --- | --- | --- | --- | --- | --- | --- |
|  |  | **Phase** | **Mean ± SE** | **Phase** | **Mean ± SE** | ***P* value** |
| **CMRO_2i_**  (x10^-10^) | <0·001 | C | 1·45 ± 0·15 | NT | 2·75 ± 0·15 | <0·001 |
|  |  | C | 1·45 ± 0·15 | RW | 1·78 ± 0·15 | 0·02 |
|  |  | NT | 2·75 ± 0·15 | RW | 1·78 ± 0·15 | <0·001 |
| **CBF_i_** (x10^-6^) | <0·001 | C | 1·90 ± 0·16 | NT | 3·53 ± 0·16 | <0·001 |
|  |  | C | 1·90 ± 0·16 | RW | 2·40 ± 0·16 | 0·004 |
|  |  | NT | 3·53 ± 0·16 | RW | 2·40 ± 0·16 | <0·001 |
| **cFTOE** | 0·001 | C | 0·28 ± 0·01 | NT | 0·30 ± 0·01 | 0·001 |
|  |  | C | 0·28 ± 0·01 | RW | 0·28 ± 0·01 | 0·70 |
|  |  | NT | 0·30 ± 0·01 | RW | 0·28 ± 0·01 | 0·003 |
| **ctSO_2_** | <0·001 | C | 71·01 ± 0·84 | NT | 67·94 ± 0·82 | <0·001 |
|  |  | C | 71·01 ± 0·84 | RW | 70·13 ± 0·84 | 0·20 |
|  |  | NT | 67·94 ± 0·82 | RW | 70·13 ± 0·84 | 0·002 |

From mixed linear model of cerebral haemodynamic measures on phase of TH, accounting for within-subject correlation within and across phases. Overall P tests H_0_:C=RW=NT. Abbreviations: C, cooled; CBF_i_, index of cerebral blood flow; cFTOE, cerebral fractional tissue oxygen extraction; CMRO_2i_, index of cerebral metabolic rate of oxygen consumption; ctSO_2_, cerebral tissue oxygen saturation; NT, normothermic; RW, rewarming.


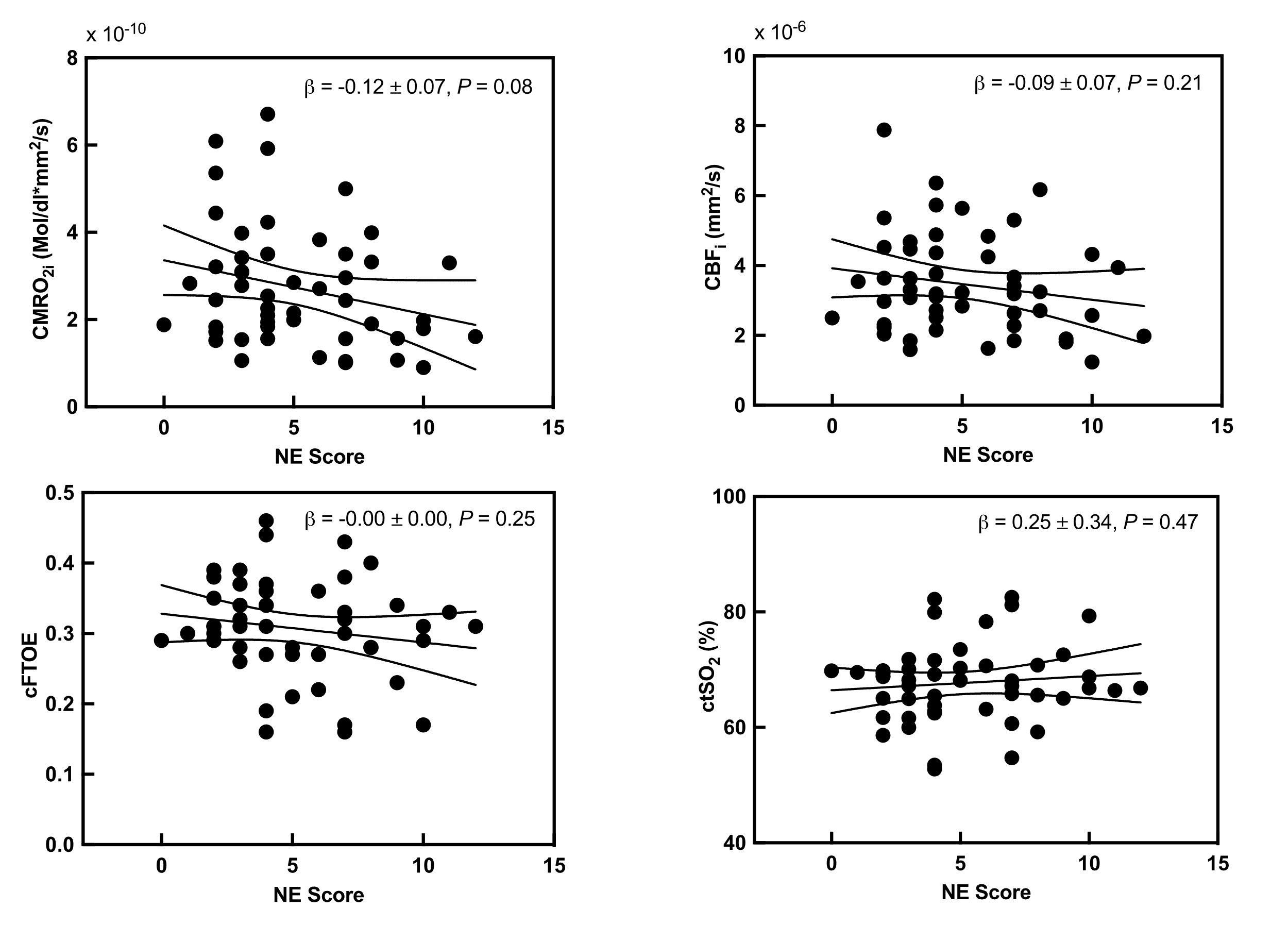
**Supplementary Figure 2. Association Between Neonatal Encephalopathy Score and Normothermic Cerebral Haemodynamic Variables**

From linear regression model of cerebral haemodynamic measures on NE score. Lines indicated fitted regression model and 95% confidence limits. P tests H_0_: β=0. Abbreviations: CBF_i_, index of cerebral blood flow; cFTOE, cerebral fractional tissue oxygen extraction; CMRO_2i_, index of cerebral metabolic rate of oxygen consumption; ctSO_2_, cerebral tissue oxygen saturation; NE, neonatal encephalopathy; β, regression coefficient ± standard error, in units of cerebral haemodynamic variable per unit change in NE score.

**Supplementary Table 4. Association of Cerebral Haemodynamic Variables with Temperature with Site, Sedatives, and Phenobarbital as Covariates**

|  | **β ± SE** | **95% CI** | ***P* value** |
| --- | --- | --- | --- |
| **Site as covariate (random effect)** | | | |
| **CMRO_2i_** (x10^-10^) | 0·38 ± 0·04 | 0·31 to 0·46 | <0·001 |
| **CBF_i_** (x10^-6^) | 0·48 ± 0·05 | 0·39 to 0·57 | <0·001 |
| **cFTOE** | 0·007 ± 0·002 | 0·00 to 0·01 | <0·001 |
| **ctSO_2_** | -0·91 ± 0·19 | -1·29 to -0·53 | <0·001 |
| **Site as covariate (fixed effect)** | | | |
| **CMRO_2i_** (x10^-10^) | 0·39 ± 0·04 | 0·31 to 0·46 | <0·001 |
| **CBF_i_** (x10^-6^) | 0·48 ± 0·05 | 0·39 to 0·58 | <0·001 |
| **cFTOE** | 0·007 ± 0·002 | 0·00 to 0·01 | <0·001 |
| **ctSO_2_** | -0·91 ± 0·19 | -1·30 to -0·53 | <0·001 |
| **Sedatives as covariate** | | | |
| **CMRO_2i_** (x10^-10^) | 0.34 ± 0.05 | 0·24 to 0·43 | <0·001 |
| **CBF_i_** (x10^-6^) | 0.44 ± 0.06 | 0·33 to 0·55 | <0·001 |
| **cFTOE** | 0.005 ± 0.002 | 0·00 to 0·01 | 0·04 |
| **ctSO_2_** | -0.83 ± 0.24 | -1·31 to -0·35 | 0·001 |
| **Phenobarbital (anti-seizure medication) as covariate** | | | |
| **CMRO_2i_** (x10^-10^) | 0·38 ± 0·04 | 0·31 to 0·46 | <0·001 |
| **CBF_i_** (x10^-6^) | 0·48 ± 0·05 | 0·39 to 0·57 | <0·001 |
| **cFTOE** | 0·007 ± 0·002 | 0·00 to 0·01 | <0·001 |
| **ctSO_2_** | -0·89 ± 0·19 | -1·28 to -0·51 | <0·001 |

From mixed linear model of cerebral haemodynamic measures on patient temperature with random intercept for patients, adjusted for indicated covariate. P tests H_0_: β=0. Abbreviations: CBF_i_, index of cerebral blood flow; cFTOE, cerebral fractional tissue oxygen extraction; CMRO_2i_, index of cerebral metabolic rate of oxygen consumption; ctSO_2_, cerebral tissue oxygen saturation; β, regression coefficient ± standard error, in cerebral haemodynamic measures per °C change in patient temperature, with 95% confidence interval.

**Supplementary Table 5.** **Association between Neonatal Encephalopathy Score and Cerebral Haemodynamic Variables**

| **Phase** | **Haemodynamic Variables** | **β ± SE** | **R^2^** | **95% CI** | ***P* value** |
| --- | --- | --- | --- | --- | --- |
| **C** | **CMRO_2i_**  (x10^-10^) | -0·03 ± 0·04 | 0·02 | -0·12 to 0·05 | 0·42 |
|  | **CBF_i_**  (x10^-6^) | -0·04 ± 0·05 | 0·01 | -0·14 to 0·06 | 0·45 |
|  | **cFTOE** | -0·00 ± 0·00 | 0·01 | -0·01 to 0·00 | 0·51 |
|  | **ctSO_2_** | 0·29 ± 0·35 | 0·02 | -0·41 to 0·99 | 0·41 |
| **RW** | **CMRO_2i_**  (x10^-10^) | 0·03 ± 0·06 | 0·01 | -0·10 to 0·16 | 0·64 |
|  | **CBF_i_**  (x10^-6^) | 0·07 ± 0·08 | 0·02 | -0·08 to 0·23 | 0·34 |
|  | **cFTOE** | -0·00 ± 0·00 | 0·02 | -0·01 to 0·00 | 0·41 |
|  | **ctSO_2_** | 0·27 ± 0·30 | 0·02 | -0·35 to 0·88 | 0·39 |
| **NT** | **CMRO_2i_**  (x10^-10^) | -0·12 ± 0·07 | 0·06 | -0·26 to 0·01 | 0·08 |
|  | **CBF_i_**  (x10^-6^) | -0·09 ± 0·07 | 0·03 | -0·23 to 0·05 | 0·21 |
|  | **cFTOE** | -0·00 ± 0·00 | 0·03 | -0·01 to 0·00 | 0·25 |
|  | **ctSO_2_** | 0·25 ± 0·34 | 0·01 | -0·43 to 0·92 | 0·47 |

From linear regression model of mean haemodynamic variable on NE score, separately by phase of TH. P tests H_0_: β=0. Abbreviations: C, cooled; CBF_i_, index of cerebral blood flow; cFTOE, cerebral fractional tissue oxygen extraction; CMRO_2i_, index of cerebral metabolic rate of oxygen consumption; ctSO_2_, cerebral tissue oxygen saturation; NT, normothermic; RW, rewarming; β, regression coefficient ± standard error, in units of cerebral haemodynamic variable per unit change in NE score, with fraction of variance explained (R^2^) and 95% confidence interval.

**Supplementary Table 6:** **Test of Group Differences in Cerebral Haemodynamic Variables Between MRI Injury Subgroups at Each Therapeutic Hypothermia Phase**

| **Haemodynamic Variables** | **Phase** | **Mean Difference (95% CI), Injury – No Injury** | ***P* value** |
| --- | --- | --- | --- |
| **CMRO_2i_**  (x10^-10^) | Cooled | 0·23 (-0·24 to 0·70) | 0·33 |
|  | Rewarming | 0·25 (-0·48 to 0·97) | 0·50 |
|  | Normothermic | -0·07 (-0·90 to 0·77) | 0·87 |
| **CBF_i_**  (x10^-6^) | Cooled | 0·41 (-0·14 to 0·95) | 0·14 |
|  | Rewarming | 0·33 (-0·55 to 1·20) | 0·46 |
|  | Normothermic | 0·20 (-0·63 to 1·03) | 0·63 |
| **cFTOE** | Cooled | -0·01 (-0·05 to 0·03) | 0·58 |
|  | Rewarming | -0·001 (-0·04 to 0·04) | 0·97 |
|  | Normothermic | -0·01 (-0·05 to 0·03) | 0·64 |
| **ctSO_2_** | Cooled | 0·58 (-3·42 to 4·58) | 0·77 |
|  | Rewarming | -0·21 (-3·82 to 3·40) | 0·91 |
|  | Normothermic | 1·42 (-2·67 to 5·52) | 0·49 |

From mixed linear model of cerebral haemodynamic measures on MRI injury subgroup, accounting for within-subject correlation. P tests H_0_:mean difference = 0. Abbreviations: CBF_i_, index of cerebral blood flow; cFTOE, cerebral fractional tissue oxygen extraction; CMRO_2i_, index of cerebral metabolic rate of oxygen consumption; ctSO_2_, cerebral tissue oxygen saturation; MRI, magnetic resonance imaging.

**Supplementary Table 7:** **Test of Group Difference in Cerebral Haemodynamic Variables Between Follow-up Subgroups at Each Therapeutic Hypothermia Phase**

| **Haemodynamic Variables** | **Phase** | **Mean Difference (95% CI), Followed – Not followed** | ***P* value** |
| --- | --- | --- | --- |
| **CMRO_2i_**  (x10^-10^) | Cooled | -0·03 (-0·48 to 0·43) | 0·91 |
|  | Rewarming | 0·02 (-0·65 to 0·68) | 0·96 |
|  | Normothermic | -0·05 (-0·83 to 0·73) | 0·90 |
| **CBF_i_**  (x10^-6^) | Cooled | -0·15 (-0·68 to 0·39) | 0·58 |
|  | Rewarming | -0·12 (-0·93 to 0·68) | 0·76 |
|  | Normothermic | 0·15 (-0·62 to 0·92) | 0·70 |
| **cFTOE** | Cooled | 0·01 (-0·03 to 0·04) | 0·80 |
|  | Rewarming | 0·02 (-0·02 to 0·05) | 0·34 |
|  | Normothermic | -0·02 (-0·06 to 0·03) | 0·45 |
| **ctSO_2_** | Cooled | -0·77 (-4·64 to 3·10) | 0·69 |
|  | Rewarming | -1·84 (-5·05 to 1·38) | 0·26 |
|  | Normothermic | 2·09 (-1·73 to 5·90) | 0·28 |

From mixed linear model of cerebral haemodynamic measures on follow-up subgroup, accounting for within-subject correlation. P tests H_0_:mean difference = 0. Abbreviations: CBF_i_, index of cerebral blood flow; cFTOE, cerebral fractional tissue oxygen extraction; CMRO_2i_, index of cerebral metabolic rate of oxygen consumption; ctSO_2_, cerebral tissue oxygen saturation.

**Supplementary Table 8.** **Association of Neonatal Variables with BSID-III Composite Scores**

| **Variables** | **BSID-III Domain** | **β ± SE** | **R^2^** | **95% CI** | ***P* value** |
| --- | --- | --- | --- | --- | --- |
| **CMRO_2i_ (NT)** (x10^-10^) | Cognitive | 4·49 ± 1·55 | 0·30 | 1·26 to 7·72 | 0·009 |
|  | Language | 2·70 ± 1·54 | 0·13 | -0·51 to 5·92 | 0·10 |
|  | Motor | 2·77 ± 1·00 | 0·28 | 0·68 to 4·86 | 0·01 |
| **CBF_i_ (NT)** (x10^-6^) | Cognitive | 4·59 ± 1·28 | 0·39 | 1·91 to 7·26 | 0·002 |
|  | Language | 1·81 ± 1·42 | 0·08 | -1·15 to 4·77 | 0·22 |
|  | Motor | 2·62 ± 0·87 | 0·31 | 0·81 to 4·44 | 0·007 |
| **cFTOE (NT)** | Cognitive | -0·73 ± 42·72 | 0·00 | -89·85 to 88·39 | 0·99 |
|  | Language | 65·27 ± 35·51 | 0·14 | -8·79 to 139·34 | 0·08 |
|  | Motor | 8·32 ± 27·23 | 0·00 | -48·47 to 65·11 | 0·76 |
| **ctSO_2_ (NT)** | Cognitive | 0·15 ± 0·43 | 0·01 | -0·75 to 1·06 | 0·73 |
|  | Language | -0·60 ± 0·37 | 0·12 | -1·36 to 0·17 | 0·12 |
|  | Motor | -0·04 ± 0·28 | 0·00 | -0·62 to 0·53 | 0·88 |
| **CMRO_2i_ (RW)** (x10^-10^) | Cognitive | 5·25 ± 2·54 | 0·21 | -0·12 to 10·63 | 0·06 |
|  | Language | 2·53 ± 2·84 | 0·05 | -3·49 to 8·56 | 0·39 |
|  | Motor | 2·24 ± 2·00 | 0·07 | -2·01 to 6·49 | 0·28 |
| **CBF_i_ (RW)** (x10^-6^) | Cognitive | 3·92 ± 2·19 | 0·17 | -0·72 to 8·57 | 0·09 |
|  | Language | 2·05 ± 2·39 | 0·04 | -3·03 to 7·12 | 0·41 |
|  | Motor | 1·43 ± 1·71 | 0·04 | -2·20 to 5·06 | 0·42 |
| **cFTOE (RW)** | Cognitive | 37·82 ± 52·19 | 0·03 | -72·82 to 148·45 | 0·48 |
|  | Language | 26·00 ± 53·69 | 0·01 | -87·82 to 139·81 | 0·64 |
|  | Motor | 27·48 ± 38·01 | 0·03 | -53·09 to 108·05 | 0·48 |
| **ctSO_2_ (RW)** | Cognitive | -0·37 ± 0·51 | 0·03 | -1·46 to 0·71 | 0·48 |
|  | Language | -0·17 ± 0·53 | 0·01 | -1·29 to 0·95 | 0·75 |
|  | Motor | -0·28 ± 0·37 | 0·03 | -1·07 to 0·51 | 0·46 |
| **CMRO_2i_ (C)** (x10^-10^) | Cognitive | 2·59 ± 2·71 | 0·07 | -3·27 to 8·44 | 0·36 |
|  | Language | 3·62 ± 3·04 | 0·10 | -2·94 to 10·18 | 0·25 |
|  | Motor | 0·67 ± 2·13 | 0·01 | -3·93 to 5·26 | 0·76 |
| **CBF_i_ (C)** (x10^-6^) | Cognitive | 3·61 ± 2·43 | 0·15 | -1·63 to 8·85 | 0·16 |
|  | Language | 2·77 ± 2·89 | 0·07 | -3·48 to 9·02 | 0·36 |
|  | Motor | 0·62 ± 1·99 | 0·01 | -3·68 to 4·92 | 0·76 |
| **cFTOE (C)** | Cognitive | -27·90 ± 43·32 | 0·03 | -121·49 to 65·70 | 0·53 |
|  | Language | 14·16 ± 50·04 | 0·01 | -93·95 to 122·27 | 0·78 |
|  | Motor | 8·15 ± 33·42 | 0·00 | -64·05 to 80·36 | 0·81 |
| **ctSO_2_ (C)** | Cognitive | 0·23 ± 0·42 | 0·02 | -0·68 to 1·15 | 0·59 |
|  | Language | -0·14 ± 0·49 | 0·01 | -1·19 to 0·91 | 0·78 |
|  | Motor | -0·21 ± 0·32 | 0·03 | -0·91 to 0·48 | 0·51 |
| **NE score** | Cognitive | -1·96 ± 1·00 | 0·16 | -4·06 to 0·13 | 0·06 |
|  | Language | -0·22 ± 0·98 | 0·00 | -2·27 to 1·83 | 0·82 |
|  | Motor | -0·81 ± 0·68 | 0·07 | -2·22 to 0·60 | 0·25 |
| **MRI injury,  Yes – No** | Cognitive | 1·56 ± 5·81 | 0·00 | -10·56 to 13·69 | 0·79 |
|  | Language | 5·00 ± 5·11 | 0·05 | -5·66 to 15·66 | 0·34 |
|  | Motor | 2·77 ± 3·67 | 0·03 | -4·88 to 10·42 | 0·46 |
| **Median Family Income, $1000** | Cognitive | 0·00 ± 0·00 | 0·01 | 0·00 to 0·00 | 0·61 |
|  | Language | 0·00 ± 0·00 | 0·10 | 0·00 to 0·00 | 0·16 |
|  | Motor | 0·00 ± 0·00 | 0·03 | 0·00 to 0·00 | 0·46 |

P tests H0: β=0. Abbreviations: C, cooled; CBF_i_, index of cerebral blood flow; cFTOE, cerebral fractional tissue oxygen extraction; CMRO_2i_, index of cerebral metabolic rate of oxygen consumption; ctSO_2_, cerebral tissue oxygen saturation; MRI, magnetic resonance imaging; NE, neonatal encephalopathy; NT, normothermic; RW, rewarming; β, regression coefficient ± standard error, in Bayley scale points per unit change in explanatory variable, with fraction of variance explained (R^2^) and 95% confidence interval.

**Supplementary Table 9. Association of Cerebral Haemodynamic Variables at Normothermia with BSID-III Composite Scores with Temperature, Neonatal Encephalopathy Scores, and Phenobarbital as Covariates**

|  | **BSID-III Domain** | **β ± SE** | **R^2^** | **95% CI** | ***P* value** |
| --- | --- | --- | --- | --- | --- |
| **Temperature as covariate** | | | | | |
| **CMRO_2i_ (NT)** (x10^-10^) | Cognitive | 4·48 ± 1·59 | 0·30 | 1·16 to 7·80 | 0·01 |
|  | Language | 2·67 ± 1·56 | 0·16 | -0·59 to 5·94 | 0·10 |
|  | Motor | 2·77 ± 1·03 | 0·28 | 0·62 to 4·92 | 0·01 |
| **CBF_i_ (NT)** (x10^-6^) | Cognitive | 4·58 ± 1·32 | 0·39 | 1·82 to 7·34 | 0·003 |
|  | Language | 1·77 ± 1·44 | 0·10 | -1·23 to 4·78 | 0·23 |
|  | Motor | 2·63 ± 0·89 | 0·31 | 0·76 to 4·50 | 0·008 |
| **cFTOE (NT)** | Cognitive | -0·87 ± 43·79 | 0·00 | -92·52 to 90·79 | 0·98 |
|  | Language | 64·84 ± 35·85 | 0·17 | -10·19 to 139·87 | 0·09 |
|  | Motor | 8·31 ± 27·94 | 0·00 | -50·16 to 66·78 | 0·77 |
| **ctSO_2_ (NT)** | Cognitive | 0·15 ± 0·44 | 0·01 | -0·78 to 1·08 | 0·73 |
|  | Language | -0·60 ± 0·37 | 0·16 | -1·37 to 0·18 | 0·12 |
|  | Motor | -0·04 ± 0·28 | 0·00 | -0·64 to 0·55 | 0·88 |
| **NE score as covariate** | | | | | |
| **CMRO_2i_ (NT)** (x10^-10^) | Cognitive | 3·83 ± 1·60 | 0·36 | 0·48 to 7·18 | 0·03 |
|  | Language | 2·87 ± 1·66 | 0·14 | -0·61 to 6·35 | 0·10 |
|  | Motor | 2·60 ± 1·08 | 0·29 | 0·35 to 4·85 | 0·03 |
| **CBF_i_ (NT)** (x10^-6^) | Cognitive | 4·06 ± 1·33 | 0·44 | 1·27 to 6·85 | 0·007 |
|  | Language | 1·90 ± 1·53 | 0·08 | -1·31 to 5·11 | 0·23 |
|  | Motor | 2·49 ± 0·94 | 0·32 | 0·53 to 4·45 | 0·02 |
| **cFTOE (NT)** | Cognitive | -7·77 ± 40·28 | 0·16 | -92·08 to 76·54 | 0·85 |
|  | Language | 65·03 ± 36·57 | 0·14 | -11·53 to 141·58 | 0·09 |
|  | Motor | 5·51 ± 27·14 | 0·07 | -51·29 to 62·31 | 0·84 |
| **ctSO_2_ (NT)** | Cognitive | 0·16 ± 0·41 | 0·17 | -0·69 to 1·01 | 0·70 |
|  | Language | -0·60 ± 0·38 | 0·12 | -1·38 to 0·19 | 0·13 |
|  | Motor | -0·04 ± 0·27 | 0·07 | -0·62 to 0·53 | 0·88 |
| **Phenobarbital (anti-seizure medication) as covariate** | | | | | |
| **CMRO_2i_ (NT)** (x10^-10^) | Cognitive | 4·59 ± 1·65 | 0·30 | 1·13 to 8·04 | 0·01 |
|  | Language | 3·54 ± 1·50 | 0·28 | 0·41 to 6·67 | 0·03 |
|  | Motor | 3·11 ± 1·03 | 0·33 | 0·95 to 5·27 | 0·007 |
| **CBF_i_ (NT)** (x10^-6^) | Cognitive | 4·57 ± 1·33 | 0·39 | 1·78 to 7·36 | 0·003 |
|  | Language | 2·16 ± 1·39 | 0·18 | -0·75 to 5·07 | 0·14 |
|  | Motor | 2·75 ± 0·89 | 0·34 | 0·89 to 4·60 | 0·006 |
| **cFTOE (NT)** | Cognitive | -6·56 ± 45·04 | 0·01 | -100·83 to 87·71 | 0·89 |
|  | Language | 82·65 ± 34·31 | 0·29 | 10·84 to 154·46 | 0·03 |
|  | Motor | 11·31 ± 28·77 | 0·01 | -48·91 to 71·52 | 0·70 |
| **ctSO_2_ (NT)** | Cognitive | 0·23 ± 0·46 | 0·02 | -0·73 to 1·18 | 0·63 |
|  | Language | -0·78 ± 0·36 | 0·26 | -1·53 to -0·03 | 0·04 |
|  | Motor | -0·07 ± 0·29 | 0·01 | -0·69 to 0·54 | 0·81 |

From linear regression model of Bayley scores on cerebral haemodynamic measures, adjusted for indicated covariate. P tests H_0_: β=0.Abbreviations: CBF_i_, index of cerebral blood flow; cFTOE, cerebral fractional tissue oxygen extraction; CMRO_2i_, index of cerebral metabolic rate of oxygen consumption; ctSO_2_, cerebral tissue oxygen saturation; NE, neonatal encephalopathy; NT, normothermic; β, regression coefficient ± standard error, in Bayley scale points per unit change in cerebral haemodynamic variable, with fraction of variance explained (R^2^) and 95% confidence interval.

**Supplementary Table 10. Sex as an Effect Modifier to the Association of Cerebral Haemodynamic Variables at Normothermia with BSID-III Composite Scores**

|  | **BSID-III Domain** | **Female β ± SE** | **Male β ± SE** | ***P* value** |
| --- | --- | --- | --- | --- |
| **CMRO_2i_ (NT)** (x10^-10^) | Cognitive | 5·16 ± 2·00 | 3·37 ± 3·01 | 0·63 |
|  | Motor | 3·30 ± 1·28 | 2·28 ± 1·92 | 0·66 |
| **CBF_i_ (NT)** (x10^-6^) | Cognitive | 4·60 ± 1·58 | 6·39 ± 3·08 | 0·61 |
|  | Motor | 2·63 ± 1·03 | 4·51 ± 2·01 | 0·42 |

From linear regression model of Bayley scores on cerebral haemodynamic measures, with sex and interaction of cerebral haemodynamic measure and sex as covariate. P tests H_0_: β=0. Abbreviations: CBF_i_, index of cerebral blood flow; CMRO_2i_, index of cerebral metabolic rate of oxygen consumption; NT, normothermic; β, regression coefficient ± standard error, in Bayley scale points per unit change in cerebral haemodynamic variable for females and males.
